# Elucidating epigenetic landscape of gastric premalignant lesions through genome‐wide mapping of 5‐hydroxymethylcytosines: A 12‐year median follow‐up study

**DOI:** 10.1002/ctm2.70114

**Published:** 2024-12-03

**Authors:** Zhongguang Luo, Wenshuai Li, Wanwei Zheng, Yixiang Shi, Maolin Ye, Xiangyu Guo, Kaiyi Fu, Changsheng Yan, Bowen Wang, Bin Lv, Shaocong Mo, Hongyang Zhang, Jun Zhang, Chuan He, Feifei Luo, Wei Zhang, Jie Liu

**Affiliations:** ^1^ Department of Digestive Diseases Huashan Hospital Fudan University Shanghai China; ^2^ Bionova (Shanghai) Medical Technology Co., Ltd. Shanghai China; ^3^ Department of Chemistry and The Howard Hughes Medical Institute The University of Chicago Chicago Illinois USA; ^4^ National Clinical Research Centre for Aging and Medicine Huashan Hospital Fudan University Shanghai China; ^5^ Department of Preventive Medicine and The Robert h. Lurie Comprehensive Cancer Center Northwestern University Feinberg School of Medicine Chicago Illinois USA

**Keywords:** 5‐hydroxymethylcytosine, cancer prevention, gastric adenocarcinoma, gastric premalignant lesions, machine learning

## Abstract

**Background:**

Epigenetic modifications are crucial in tumourigenesis, yet the changes in novel epigenetic regulators like 5‐hydroxymethylcytosines (5hmC) during the evolution of gastric premalignant lesions remain poorly understood. This study aims to investigate the implications of 5hmC in the progression from gastric premalignant lesions to gastric adenocarcinoma (GAC).

**Methods:**

To our knowledge, we conducted the largest and longest longitudinal study of a Chinese population with gastric precursor lesions, involving 29,176 patients with gastritis who underwent gastroscopy and biopsy between 2001 and 2015, with follow‐up until 1 August, 2022. The median follow‐up time was 12.2 years, and the overall GAC incidence rate was 0.82%. Genome‐wide mapping of 5hmC in gastric premalignant lesions from a subset of individuals was performed using the 5hmC‐Seal assay, including 21 samples that progressed to GAC during follow‐up and 48 non‐progressed age‐ and sex‐matched controls.

**Results:**

We identified 213 differentially modified gene bodies, primarily concentrated in pathways related to cell division, cell cycle, energy metabolism, inflammation and tumourigenesis. An exploratory study was conducted to summarize a 5hmC‐based epigenetic model for predicting cancer progression using multivariable logistic regression and machine learning. The nine‐gene model showed an area under the curve of 87.5% (95% confidence interval: 72%–100%) in the validation samples (one of three), which were set aside before model training.

**Conclusions:**

This study is the first to explore the 5hmC molecular landscape in gastric premalignant lesions, suggesting relevant pathways implicated in their evolution to GAC as well as the feasibility of exploiting genome‐wide 5hmC mapping in assessing the risk of future cancer progression.

**Key points:**

- A largest longitudinal follow‐up study of gastric precursor lesions in Chinese patients.
- Revealing novel 5hmC molecular landscape linked to gastric premalignant lesions.
- The feasibility of an innovative 5hmC‐based predictive model for assessing gastric cancer progression risk.

## Graphical abstract

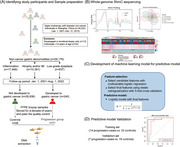

## 1 INTRODUCTION

Gastric cancer (GC) remains a substantial healthcare challenge globally with considerable implications for public health. In 2020, stomach cancer claimed around 768,793 lives worldwide, accounting for approximately 7.7% of all cancer‐related deaths.^1^ The global mortality burden of stomach cancer is remarkably similar to its incidence, with East Asia having the highest burden.^1^ In particular, China reports the highest incidence, primarily of the adenocarcinoma type (90%–95%) accounting for more than 40% of global cases.^2^ The precursor lesions of gastric adenocarcinoma (GAC), including gastric atrophy, intestinal metaplasia (IM) and dysplasia, hold particular significance due to the substantial healthcare burden imposed by their high‐risk progression to GAC. Understanding molecular factors contributing to this transition is urgently needed with high clinical impact,^3^, ^4^, ^5^ because timely detection of these premalignant stages could be crucial for improved treatment outcomes for GAC. In current clinical practice, endoscopic evaluation systems such as magnified endoscopy or narrow‐band imaging, coupled with histological assessments based on random biopsies, are commonly used to assess the severity of premalignant lesions using such grading systems as the Operative Link on Gastric Intestinal Metaplasia (OLGIM) and the Operative Link on Gastritis assessment (OLGA).^6^ Although a large‐scale retrospective study found that the high‐risk OLGA and OLGIM stages in primary endoscopy predicted risk of Ggastric cancer with sensitivities of 21% and 32% of cancer cases, respectively,^7^ it is clear that there is still a need to further improve the predictive accuracy for early‐stage GAC.

The development of GAC in individuals with premalignant lesions is a complex and multifaceted process shaped by the intricate interplay between multiple genetic and non‐genetic factors. Although the role of genetics in GAC susceptibility has been acknowledged, genetics alone cannot fully explain the risk. Considering its complex nature, the contribution of non‐genetic factors, such as epigenetic modifications, *Helicobacter pylori* infection, environmental exposure, dietary habits and intestinal microbiota to this transition, remains subjects of intensive investigation. Previous epidemiological studies have shown that the high‐risk factors for GC include age, gender, gastric pathology type, race, *H. pylori* infection, serum pepsinogen I/II ratio, gastrin‐17 levels and so on. Epigenetic mechanisms, such as cytosine modifications, histone modifications and non‐coding RNAs, reveal the information encoded in the genome with all the molecular and chemical cues of cellular, extracellular and environmental origins, contributing to gene regulation of each cell type to define its functional identity during normal biological processes and development as well as disease pathogenesis.^8^ In the context of precancerous gastric lesions, known risk factors like chronic inflammation caused by *H. pylori* infection, genetic predisposition, poor dietary choices, lifestyle behaviour, environmental exposure and viral infections, such as the Epstein–Barr virus, can trigger epigenetic alterations,^9^, ^10^ which in turn may disrupt normal cellular regulation, promote abnormal cell proliferation and ultimately lead to tumourigenesis.^11^, ^12^, ^13^ Understanding the epigenetic landscape of gastric precancerous lesions may improve our understanding the mechanism underlying their transition to GAC and help identify novel therapeutic/intervention targets and diagnostic/predictive biomarkers as well. Previous studies have indicated that the methylation status of CpG islands in promoter regions plays a crucial role in the development of GAC.^14^, ^15^, ^16^ It is known that hypermethylation in promoters of tumour suppressors is often associated with the loss of gene expression, which contributes to tumourigenesis and progression.^17^ However, majority of previous studies were limited by epigenetic profiling techniques that could not distinguish the more abundant 5‐methylcytosines (5mC) from 5‐hydroxymethylcytosines (5hmC), the biochemically stable product of active demethylation of 5mC with a distinct genomic distribution and gene regulatory role. While 5hmC modifications have emerged to be clinically relevant biomarkers for a variety of human cancers and diseases,^18^, ^19^, ^20^, ^21^, ^22^, ^23^ to our knowledge, the specific involvement of 5hmC in the aggressiveness of precancerous lesions that transition to GAC has not been characterized.^24^, ^25^, ^26^ Specifically, for human cancers, loss of 5hmC has been linked to reduced expression of tumour suppressor genes and genomic instability,^23^ and alterations in 5hmC can impact metabolic pathways and inflammatory responses, underscoring its significance in disease pathogenesis.^21^, ^22^, ^27^ ^,28^ As a marker of active gene expression, 5hmC can influence transcription factor binding and chromatin accessibility, making these modified cytosines key players in the epigenetic landscape to be explored for their potential as novel biomarkers or therapeutic targets.

Taking advantage of our unique clinical resources and the availability of tissue samples as well as the robust 5hmC‐Seal technique for genome‐wide 5hmC mapping,^14^, ^21^ which was developed and optimized by our team for clinical biospecimens,^24^ the current study aims to unravelling the impact of 5hmC in shaping the transition from premalignant gastric lesions to GAC. Our objectives are twofold: firstly, to assess the incidence of GAC among patients with gastric atrophy, IM, or dysplasia and to compare their relative risks for GAC with the inflammation population; and second, to characterize the 5hmC landscape in gastric precancerous lesions and assess the clinical outcomes of gastric premalignant lesions by evaluating the feasibility of constructing a 5hmC‐based epigenetic model for predicting cancer progression.

## 2 METHODS

### 2.1 Setting

This retrospective cohort study was conducted between 2001 and 2015 targeting residents of Shanghai, China, who were treated at Huashan Hospital, Fudan University.

### 2.2 Identifying study participants

**FIGURE 1:**
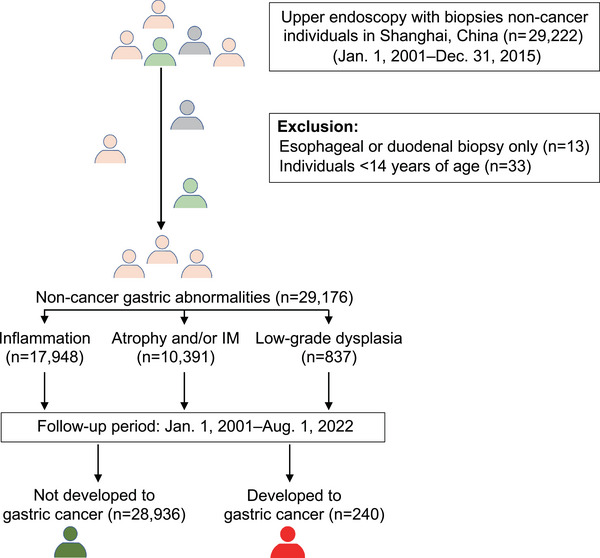
Study design and overview of workflow. Follow‐up of patients with gastric inflammation, atrophy, intestinal metaplasia and dysplasia develop to GAC premalignant lesions.

We identified 29,176 patients who underwent gastric biopsy at Huashan Hospital, Fudan University between 2001 and 2015 and were followed up until 1 August, 2022. The study design and overview of workflow are shown in Figure 1. Furthermore, GAC cases diagnosed between 2000 and 2022 were identified by the Shanghai Center for Disease Control (CDC). The diagnostic criteria for gastritis relied on the Consensus for Diagnostic Pathway in Biopsies of Chronic Gastritis and Epistemic Neoplasms in the 2010 World Health Organization guidelines and Classification and Grading of Gastritis: The updated Sydney System International Workshop on the Histology of Gastritis, Houston 1994.^29^, ^30^, ^31^

### 2.3 Sample preparation and genomic DNA isolation

An initial search of 240 biopsy samples that had undergone cancer transformation identified 25 individuals had retrievable formalin‐fixed paraffin‐embedded (FFPE) biospecimens. Based on the 1:2 case–control ratio, we randomly matched control samples that did not progress to cancer and were similar in age, gender and biopsy time to the progressed samples. Genomic DNA (gDNA) was extracted from the FFPE tissue samples using the Qiagen GeneRead DNA FFPE Kit according to the standard procedures, followed by ultrasound fragmentation. Only gDNA samples with at least 15 ng at a concentration of >1.5 ng/µL were retained for genome‐wide 5hmC profiling. The DNA fragments were approximately 350 bp in size using gel electrophoresis, and showed no degradation peaks greater than 1000 bp. In total, 69 samples were selected for genome‐wide 5hmC profiling after quality control, including 21 samples of patients progressed (“progression group”) into GAC, and 48 patients did not progress after at least 18 years of follow‐up (“controls”). The clinical characteristics of the 69 gastric biopsy samples (inflammation: *n* = 32, atrophy and/or IM: *n* = 26 and low‐grade dysplasia: *n* = 11, Figure 4A) are shown in Table S3. At 80% statistical power, we estimated to detect differential features with 0.05 type‐I error, representing moderate to large effect sizes (0.783).

### 2.4 Library construction for 5hmC profiling, sequencing and bioinformatic processing

Detailed protocols for 5hmC‐Seal library construction, sequencing and bioinformatic processing were described in our previous publications.^24^, ^32^, ^33^ Briefly, the fragmented gDNA samples were prepared and ligated to adaptors. Subsequently, the T4 bacteriophage enzyme β‐glucosyltransferase was used to transfer an engineered glucose moiety containing an azide group to 5hmC in duplex DNA. A biotin tag was installed onto the azide group using Huisgen cycloaddition (“Click”) chemistry, followed by capturing 5hmC‐containing DNA fragments using avidin beads. The 5hmC‐Seal library was then constructed using PCR amplification, followed by paired‐end sequencing (PE150) on the Illumina Next‐Seq 500 platform at Mingma Technologies Co., Ltd.

Raw sequencing reads were trimmed and filtered for low‐quality bases using the FASTX Toolkit (v0.0.14), followed by alignment to the human genome reference (hg19) using Bowtie2 (v2.2.6) in the end‐to‐end alignment mode.^34^ Read pairs were concordantly aligned with fragment length ≤500 bp and with up to one ambiguous base and up to four mismatched bases per 100 bp in length. The aligned read pairs were sorted, indexed and deduplicated using Picard (v2.6.0) (http://broadinstitute.github.io/picard).

### 2.5 Differential analysis for 5hmC signatures

Raw read counts were normalized using DESeq2,^35^ which performs internal normalization to correct the library size. The ComBat tool was used to minimize batch effects and control unwanted sources of variation in the genome‐wide 5hmC‐Seal data.^36^ In addition to gene bodies, the 5hmC‐Seal data were summarized based on *cis*‐regulatory elements, specifically promoter regions, and histone modification marks (H3K4me1 and H3K27ac) for enhancers derived from various tissues, including lung, brain, liver and heart, provided by the Roadmap Epigenomics Project according to the start and end annotations of the gapped peaks.^37^ The Benjamini–Hochberg procedure was used to control false discovery rate (FDR) in differential analysis.^38^

### 2.6 Functional exploration

The Gene Ontology and Kyoto Encyclopedia of Genes and Genomes (KEGG) databases were used to explore the functional relevance of the differentially modified gene bodies, promoters and histone modification/enhancer markers.^39^, ^40^, ^41^ The Benjamini–Hochberg method was used to control FDR (5%) for significant pathways and biological processes. Empirical *p*‐values were calculated using the wilcox_test in R *coin* package, which divided regular *p*‐value by 2. Gene set enrichment analysis (GSEA) was conducted using the R package *clusterProfiler* (version 4.4.4).^42^

### 2.7 Statistical modelling for cancer progression risk

The patients that progressed to GAC (*n* = 21) and controls (*n* = 48) were randomly grouped into the training (2/3) and internal validation (1/3) sets (Figure 4D). Feature selection was performed in the training set using the elastic net regularization on the multivariable logistic regression model, starting from a list of the most informative candidate gene bodies (nominal *p*‐value < 0.01) from differential analysis using logistic regression controlled for age and gender between cases and controls. Specifically, the elastic net‐logistic regression model was cross‐validated (fivefold) for a grid of parameter values for α (0.05–1.0, controlling the relative proportion of the Ridge and Lasso penalty) and λ (10^−5^–1.0, controlling the overall strength of penalty). This selection process was repeated 100 times by bootstrapping, and the top‐ranked features (i.e., importance value > 20 in each iteration) selected in at least 80% of the iterations were retained to fit the final multivariable logistic model. Receiver operating characteristic curves and 95% confidence intervals (CIs) were generated to evaluate the model performance. A cutoff value to maintain at least 90% specificity in the validation set was used to evaluate the sensitivity and specificity of all samples. We also evaluated model performance through multivariable analysis by considering various clinical/demographical variables, including gender, age and gastric lesion type. All statistical tests were performed under the R Statistical Environment.^43^

### 2.8 Statistical analysis of epidemiological data

Descriptive statistics, including frequencies, proportions and medians, were employed to characterize the demographic and clinical features of the cohort, such as age at initial endoscopy, age at GAC diagnosis, gender, histopathology and biopsy location. To assess the relative risks for the development of GAC based on various predictors (e.g., gender, age and histopathology), Cox regression analyses were performed to calculate hazard ratios (HRs). Statistical analyses were conducted using SPSS v27, with nominal *p*‐values of less than 0.05 deemed statistically significant. A nomogram, which is a graphical representation that provides a visual tool for Cox regression and calculating the probability of a particular outcome (i.e., probability of cancer transformation) based on various predictor variables (e.g., gender, age and histopathology), was generated using the R Statistical Environment.^44^ The standardized incidence ratio (SIR) was calculated to compare the estimated relative risk with the general population of China.^45^ The SIR was determined by dividing the observed number of GAC cases by the expected number of cases, which was computed by multiplying the age‐gender‐calendar year‐specific GAC incidence rates of the Chinese population by the corresponding counts in the cohort and summing the results.

## 3 RESULTS

### 3.1 Follow‐up outcomes of patients with premalignant lesions

**FIGURE 2:**
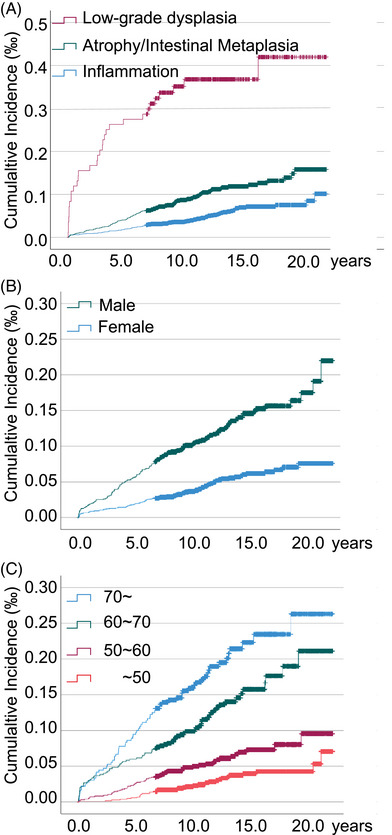
Follow‐up of patients with premalignant lesions. (A–C) The Kaplan–Meier method was used to plot cumulative incidence of gastric adenocarcinoma in patients with premalignant gastric lesions pathology, age, and sex with 20 years follow‐up.

The median follow‐up time for the 29,176 patients who underwent gastric biopsy was 12.2 years (ranging from 1.02 to 21.59 years), with a total of 370,561.99 person‐years. Pathologically, 17,948 patients had inflammation, 10,391 had atrophy and/or IM and 837 had low‐grade dysplasia. Each case was characterized by gender, affected location (i.e., antrum, corpus, cardia, fundus, angle, or unspecified), age (i.e., < 50‐, 50–59‐, 60–69‐, > 70 years old) and tissue pathology (i.e., inflammation, atrophy and/or IM and low‐grade dysplasia) (Table 1). A total of 240 cases of GAC were reported during the follow‐up period, with an overall incidence rate of 0.82% (Table 2). Figure 2A–C shows the cumulative incidence rates of GAC according to lesion type, gender and age, respectively. Specifically, 11 (0.061%) new cases of GAC were diagnosed within the first year of the index endoscopy out of the 17,948 patients with inflammation, with 86 (0.48%) additional cases of GAC diagnosed in the following years. The median time required for inflammation to progress to GAC was 6.68 years (interquartile range [IQR]: 1.02–20.62 years). Out of the 10,391 patients with atrophy and/or IM, 11 (0.11%) cases of GAC were diagnosed within the first year of the index endoscopy and 101 (0.97%) additional GAC patients diagnosed in the following years. The median time required for progression from atrophy and/or IM to GAC was 6.14 years (IQR: 1.08–18.93 years). In comparison, out of the 837 patients with low‐grade dysplasia, 13 patients (1.55%) developed GAC within the first year of diagnosis and 18 (2.15%) additional GAC cases diagnosed in the following years, with a median time of progression of 4.11 years (IQR: 1.89–15.91 years).

**TABLE 1:** Characteristics of the study participants at baseline endoscopy.

Number of patients, *n* (%) 29,176	Inflammation, 17,948	Atrophy and/or intestinal metaplasia (IM), 10,391	Low‐grade dysplasia, 837
Gender			
Male, *n* (%) 11,915 (40.84)	7009 (39.05)	4412 (42.46)	494 (59.02)
Female, *n* (%) 17,261, (59.16)	10,939 (60.95)	5979 (57.54)	343 (40.98)
Age group, *n* (%)			
<50, 10,083 (34.56)	7510 (41.84)	2393 (23.03)	180 (21.51)
50–59, 9511 (32.60)	5584 (31.11)	3656 (35.18)	271 (32.38)
60–69, 5988 (20.52)	3147 (17.53)	2622 (25.23)	219 (26.16)
70+, 3594 (12.32)	1701 (9.51)	1707 (16.55)	167 (19.95)
Location of pathology, *n* (%)			
Antrum, 26,864 (92.08)	16,549 (92.08)	9612 (92.50)	703 (83.99)
Corpus, 1004 (3.44)	744 (4.15)	211 (2.03)	49 (5.85)
Cardia, 98 (0.34)	73 (0.41)	20 (0.19)	5 (0.60)
Fundus, 251 (0.86)	234 (1.30)	15 (0.14)	2 (0.24)
Angle, 916 (3.14)	317 (1.76)	522 (5.02)	77 (9.20)
Unclear, 43 (0.15)	31 (0.17)	11(0.10)	1(0.12)

**TABLE 2:** Follow‐up outcomes of patients with gastric inflammation, atrophy and/or intestinal metaplasia (IM) and dysplasia diagnosis of gastric adenocarcinoma.

Number of patients 29,176, *n* (%)	Inflammation 17,948 (%)	Atrophy and/or IM 10,391 (%)	Low‐grade dysplasia 837 (%)
Total number of GAC, *n* (%) 240 (0.82)	97 (0.54)	112 (1.08)	31 (3.70)
Number of GAC during first year, *n* (%) 35 (0.12)	11 (0.06)	11 (0.11)	13 (1.55)
Number of GAC after first year, *n* (%) 205 (0.7)	86 (0.48)	101 (0.97)	18 (2.15)
Age at diagnosis of GAC 240			
Median	65	70	70
IQR range, years	28–94	49–94	51–90
Time to diagnosis of GAC (excluding cases during first year), years			
Median	6.68	6.14	4.11
IQR range, years	1.02–20.62	1.08–18.93	1.89–15.91
Time to diagnosis of GAC (all cases), years			
Median	6.47	5.74	2.54
IQR range, years	(0.09–20.62)	(0.13–18.93)	(0.09–15.91)
Gender			
Male (151)	59 (0.33)	69 (0.66)	23 (2.758)
Female (89)	38 (0.21)	43 (0.41)	8 (0.96)
Age at diagnosis of GAC (years)			
<50 (35)	21 (0.12)	13 (0.13)	1 (0.12)
50–59 (43)	22 (0.12)	17 (0.16)	4 (0.48)
60–69 (93)	32 (0.18)	47 (0.45)	14 (1.67)
70+ (69)	22 (0.12)	35 (0.34)	12 (1.43)

Abbreviations: GAC, gastric adenocarcinoma; IQR, interquartile range.

Overall, the gender ratios were similar (chi‐square test *p* > 0.05) between patients with inflammation (0.33% males vs. 0.21% females) and those with atrophy and/or IM (0.66% males vs. 0.41% females) who developed GAC. However, a significantly higher (chi‐square test *p* < 0.01) proportion of males (2.75%) with low‐grade dysplasia progressed to cancer at the index endoscopy than females (0.96%).

### 3.2 Evaluating risk factors for progression to GAC among patients with gastric premalignant lesions

We sought to investigate predictors of progression from premalignant gastric lesions to GAC, including gender, age, histopathology and the location of gastric abnormalities (Table 3). The univariate tests showed no statistically significant differences in the risk of cancer progression regarding premalignant locations. Gender, age and severity of histological changes were significantly associated with cancer progression. Overall, female patients showed a decreased risk (HR = 0.395, 95% CI = 0.304–0.513, *p*‐value < 0.001) compared to male patients, regardless of the lesion type. Individuals ≥70 years old (HR = 6.088, 95% CI = 4.055–9.141, *p*‐value < 0.001), 60–69 years old (HR = 4.136, 95% CI = 2.772–6.171, *p*‐value < 0.001) and 50–59 years old (HR = 1.886, 95% CI = 1.239–2.870, *p*‐value = 0.003) had a higher risk than those <50 years old. Moreover, low‐grade dysplasia showed a substantially higher risk of progression to GAC compared to inflammation alone (HR = 7.049, 95% CI = 4.705–10.563, *p*‐value < 0.001), whereas atrophy and/or IM exhibited an average 1.99‐fold increased risk compared to inflammation alone (95% CI = 1.519–2.616, *p*‐value < 0.001) (Table 3).

**TABLE 3:** Risk factors for progression to gastric adenocarcinoma among patients with gastric premalignant lesions.

	Univariate		Multivariate	
	HR (95% CI)	*p*‐value	HR (95% CI)	*p*‐value
Gender				
Male	1		1	
Female	0.395 (0.304–0.513)	<0.001	0.462 (0.354–0.602)	<0.001
Age (years)				
<50	1		1	
50—59	1.886 (1.239–2.870)	0.003	1.781 (1.167–2.718)	0.007
60—69	4.136 (2.772–6.171)	<0.001	3.516 (2.345–5.271)	<0.001
70+	6.088 (4.055–9.141)	<0.001	4.716 (3.117–7.134)	<0.001
Histopathology				
Inflammation	1		1.0	
Atrophy and intestinal metaplasia (IM)	1.994 (1.519–2.616)	<0.001	1.559 (1.183–2.055)	0.002
Dysplasia	7.049 (4.705–10.563)	<0.001	4.702 (3.119–7.089)	<0.001
Location of gastric abnormalities				
Fundus	1			
Non‐fundus	0.784 (0.195–3.153)	0.874		

Abbreviation: CI, confidence interval; HR, hazard ratio.

**FIGURE 3:**
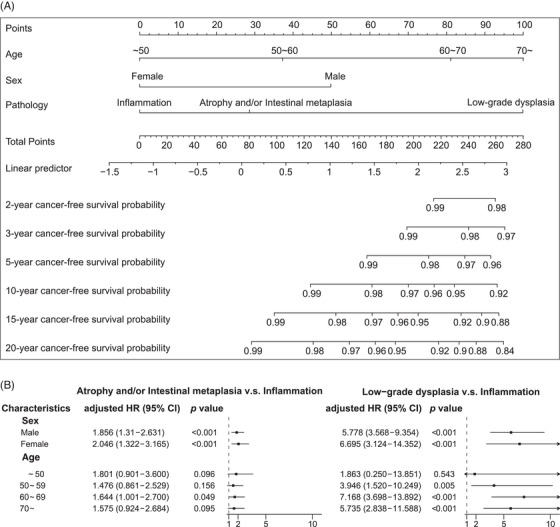
Risk factors for progression to GAC among patients with gastric premalignant lesions. (A) Instructions for using the nomogram. Draw a line perpendicular from the corresponding axis of each risk factor until it reaches the top line labelled “POINTS.” Sum up the number of points for all risk factors then draw a line descending from the axis labelled “TOTAL POINTS” until it intercepts each of the survival axes to determine 2‐, 3‐, 5‐, 10‐, 15‐, and 20‐year cancer‐free survival probabilities. (B) Stratifying the study population based on sex and age to investigate the cancer risk associated with different pathological subtypes in different sex and age groups.

Multivariable regression analyses that considered gender, age and premalignant type together further confirmed that female patients had a decreased risk (HR = 0.462, 95%, CI = 0.354–0.602, *p*‐value < 0.001) compared to male patients, after controlling other risk factors as well. Male patients with premalignant gastric lesions had a higher risk of developing GAC than female patients. Individuals aged ≥70 (HR = 4.716, 95% CI = 3.117–7.134, *p*‐value < 0.001), 60–69 (HR = 3.516, 95% CI = 2.345–5.271, *p*‐value < 0.001) and 50–59 years old (HR = 1.781, 95% CI = 1.167–2.718, *p*‐value = 0.007) consistently exhibited a higher risk of progression to GAC compared to those aged <50 years old under the multivariable analysis. Furthermore, multivariable analysis indicated that low‐grade dysplasia showed a significantly higher risk of progression to GAC compared to the presence of inflammation only (HR = 4.702, 95% CI = 3.119–7.089, *p*‐value < 0.001), and the same trend was retained for atrophy and/or IM against inflammation (HR = 1.559, 95% CI = 1.183–2.055, *p*‐value = 0.002) (Table 3). Based on these findings, a nomogram was configured (Figure 3A). Subsequently, a GAC prediction model incorporating age, gender and pathological type was constructed, achieving an area under the curve (AUC) of 72.7% (with 69.2% sensitivity and 67.8% specificity) (Figure S1).

### 3.3 Risk of GAC development stratified by age and gender

Furthermore, we stratified the study population based on gender and age to investigate the cancer risk associated with different pathological subtypes in different gender and age groups. When stratified by gender, males and females with low‐grade dysplasia and atrophy and/or IM showed a significantly higher risk of progression to GAC than those with inflammation alone. When stratified by age groups, the presence of premalignant gastric lesions did not significantly affect the risk of GAC progression in the group of individuals aged <50 years old. However, the risk of GAC was notably elevated in older age groups, especially after the diagnosis of more severe lesions at the baseline. Of note, the 60–69 years age group exhibited a particularly high risk of progression to GAC when associated with low‐grade dysplasia (Figure 3B).

### 3.4 Risk of developing GAC within 10 years

Furthermore, an analysis on the 10‐year follow‐up period by including 16,198 patients who underwent gastric biopsy at Huashan Hospital, Fudan University between 2001 and 2010 was conducted. These individuals were followed up until 1 January, 2011. Pathologically, 9922 had inflammation, 5808 had atrophy and/or IM and 468 had low‐grade dysplasia. The overall incidences of GAC in patients with premalignant lesions were 51, at an incidence rate of 0.36% during the 10‐year follow‐up period (Table S1). The cumulative incidence rates of GAC among the three pathological groups are shown in Figure S2. Additionally, multivariate Cox regression analysis showed that the male gender, age over 70 years old at initial diagnosis and low‐grade dysplasia were independently associated with an elevated risk of progress to GAC within 10 years (Table S2).

### 3.5 Differential 5hmC signatures associated with GAC progression from premalignant lesions

**FIGURE 4:**
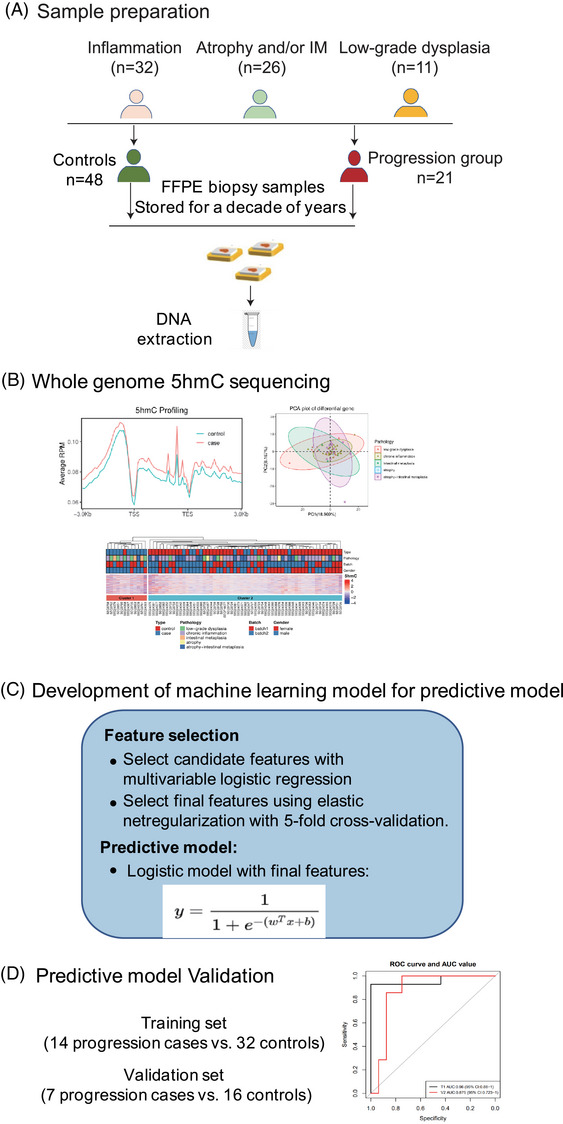
The workflow of characterizing differentially 5‐hydroxymethylcytosines (5hmC) expressed genes in the progression group versus control group. (A) In total, 69 samples were selected for genome‐wide 5hmC profiling after quality control, including 21 samples from patients who progressed to gastric cancer (GC) (“progression group”), and 48 samples from patients who did not progress after at least 18 years of follow‐up (“controls”). (B–D) Shown is an overview of the technical and analytical pipeline of the study, which includes DNA collection and preparation, library construction for 5hmC profiling, sequencing, and bioinformatic processing, feature selection and modelling using a machine‐learning approach, and validation of model performance.

**FIGURE 5:**
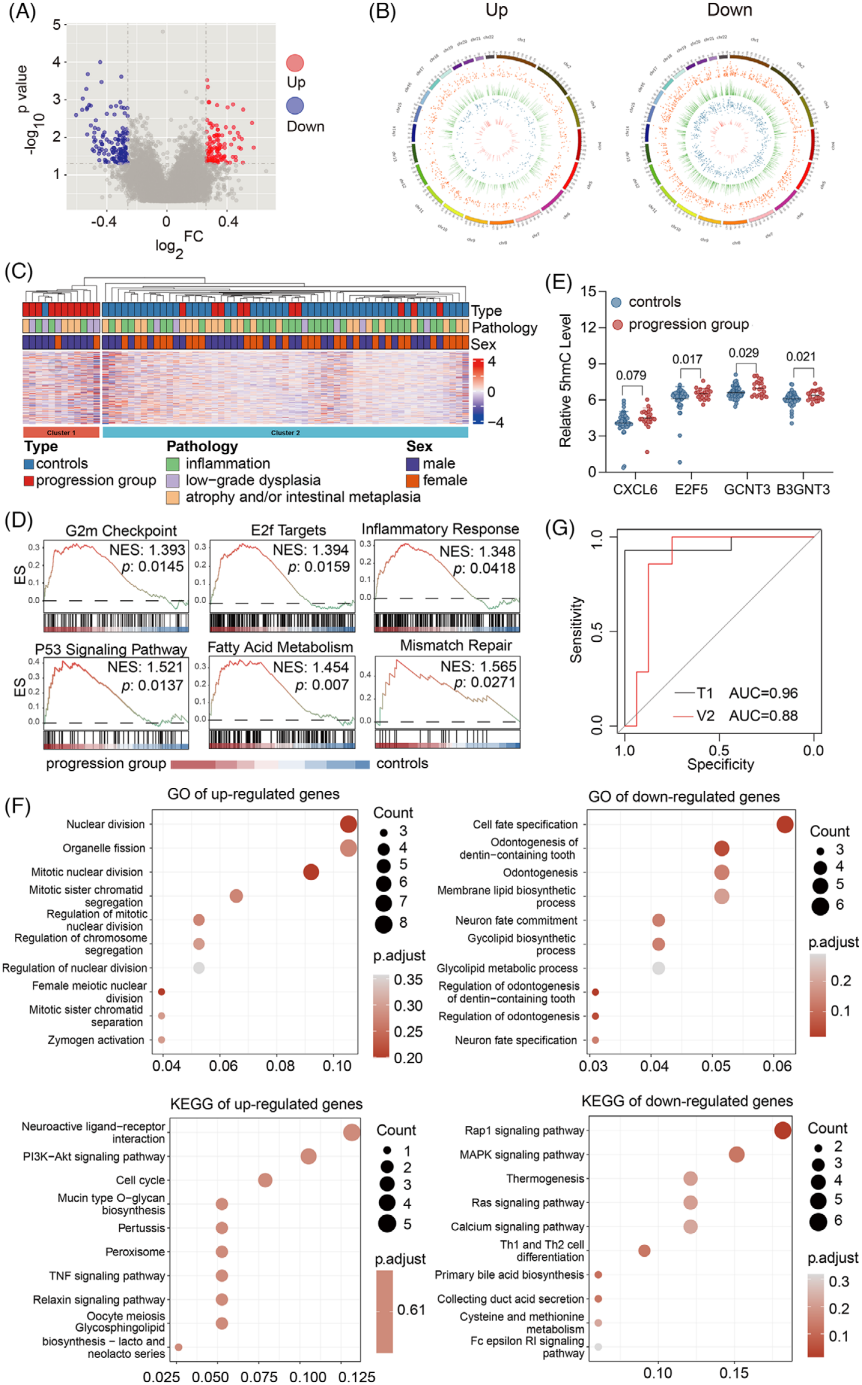
Differentially 5‐hydroxymethylcytosines (5hmC) expressed genes in the progression group versus control group. (A) Volcano plot showing the up‐regulated genes (red), and the down‐regulated genes (blue) with a |log_2_FoldChange| > 0.26, and with *p* < 0.05. (B) Genomic distributions of differentially hydroxymethylated features (false discovery rate [FDR] < 0.05 and fold‐change > 20%) between progression group and control group. Up: up‐modified; Down: down‐modified. Inner to outer circles: genebody (red), promoter (blue), H3k27ac (green), and H3k4me1 (orange), respectively. (C) Hierarchical clustering of samples based on differentially hydroxymethylated features (FDR < 0.05 and fold‐change > 20%) between the progression group and control group. (D) Representative pathways enriched in the identified genes of differentially modified between progression group and control group in gene body as determined by gene set enrichment analysis (GSEA). (E) The differentially modified 5hmC levels of different genes between progression group and control group. (F) The Kyoto Encyclopedia of Genes and Genomes (KEGG) pathway analysis and the Gene Ontology (GO) enrichment analysis of differentially modified 5hmC between progression group and control group in gene bodies of genes. (G) Classification of training and validation sets using 5hmC classifier, sex, age, and gastric lesion type.

Out of the 69 samples subjected to the 5hmC‐Seal assay and sequencing, there were 21 in the progression group and 48 in the non‐progression (control) group. We further analyzed differential 5hmC modifications between the two groups and construct a predictive multivariable model for cancer progression (Figure 4B–D). We observed potential age and gender differences between the progression and control groups; therefore, further analyses were matched for gender and age (Figure 3A,B). Principal components analysis indicated no systemic bias or batch effects in the genome‐wide 5hmC‐Seal data. The summarized genome‐wide 5hmC profiles showed enrichment in genomic regions, particularly gene bodies between the transcription start (TSS) and transcription end sites (TES) relative to their flanking regions and depletion at promoter regions around the TSS (Figure S3C–E). The Volcano plot showed differentially modified 5hmC features (gene bodies) were present between cases and controls. Specifically, a total of 213 differentially modified gene bodies were identified between the progression group and controls using DESeq2 (|log_2_FoldChange| > 0.26, empirical *p*‐value < 0.05). At this statistical cut‐off, 95 gene bodies were hyperhydroxymethylated and 118 hypohydroxymethylated in the progression group (Figure 5A). Most of these differential 5hmC features (91%–99%) appeared to have lower modification levels in the progression group relative to the controls (Figure 5B). Hierarchical clustering of samples based on differentially hydroxymethylated features (FDR < 0.05, fold‐change >20%) is shown (Figure 5C). In comparison, 1073 promoter regions (*p* < 0.05, |log_2_FoldChange| > 0.26) were differentially hydroxymethylated, with 237 showing hyper‐hydroxymethylation and 836 showing hypo‐hydroxymethylation in the progression group. Moreover, there were 2955 differentially modified H3K27ac loci (917 hyper‐hydroxymethylated and 2038 hypo‐hydroxymethylated in the progression group) and 2294 differentially modified H3K4me1 loci (804 hyper‐hydroxymethylated and 1490 hypo‐hydroxymethylated in the progression group). Hierarchical clustering of samples based on differentially hydroxymethylated features (FDR < 0.05, fold‐change > 20%) between the progression group and controls is shown for different genomic feature types (Figure S3F–H). Notably, the GSEA results indicated that the genes annotated by these differentially modified 5hmC features were associated with such pathways and biological processes as cell division, cell cycle, energy metabolism, inflammation and tumourigenesis, including the G2 M checkpoint, E2F targets, inflammatory response, p53 pathway, fatty acid metabolism and mismatch repair (Figure 5D). Specifically, some known oncogenes showed higher modification levels in the progression group, including *E2F5*, *GCNT3* and *B3GNT3* (Figure 5E). Interestingly, the highly modified chemokine *CXCL6* was previously suggested to be associated with local immune suppression and promotion of tumour progression in the immune microenvironment (Figure 5E). Furthermore, we found in the TCGA database that the expression levels of these four genes were highly upregulated in GC patients, thus positively correlated with the modification levels of 5hmC features (Figure S4).

We performed functional analysis in the gene bodies based on the differentially modified 5hmC. The top enriched KEGG pathways included, for example, mucin type O‐glycan biosynthesis, the tumor necrosis factor (TNF) signalling pathway and the phosphatidylinositol‐3kinase/protein kinase B (PI3K/Akt) signalling pathway (Figure 5F).^46^, ^47^ Furthermore, we analyzed the differential 5hmC loci in the promoter, H3K4me1 and H3K27ac regions, and conducted functional analysis between the two groups (Figure S3I–K).

### 3.6 Exploring the feasibility of a 5hmC‐based predictive model for cancer progression

Considering that gender, age, gastric lesion type and differential 5hmC levels were high‐risk factors for gastric carcinogenesis, we evaluated whether it was feasible to construct a predictive multivariable model for cancer progression (Figure 4C,D). By applying the elastic net regularization to candidate gene bodies using logistic regression modelling, a panel of nine genes (i.e., *GATA6, SLC4A3, CBARP, KCNS3, MUC5AC, CRACR2A, ZDHHC11B, ANKAR* and *SHANK3*) were identified. Five other important factors, that is, gender, age, gastric lesion type, as well as HRs between gender, age and gastric lesion types, were also added into the final model. In the training set (14 progression cases vs. 32 controls), the model showed a high capacity for identifying patients who later progressed to GAC from controls at an AUC of 96% (95% CI: 88.0%–100%) (Figure 5G). Comparable performance was achieved in the validation set (7 progression cases vs. 16 controls) with an AUC of 87.5% (95% CI: 72.3%–100%). In all testing samples, the prediction algorithm achieved a sensitivity of 90.5% (95% CI: 68.1%–98.3%) with a specificity at 93.8% (95% CI: 81.8%–98.4%).

### 3.7 Characterization differential 5hmC associated with different type of gastric premalignant lesions

**FIGURE 6:**
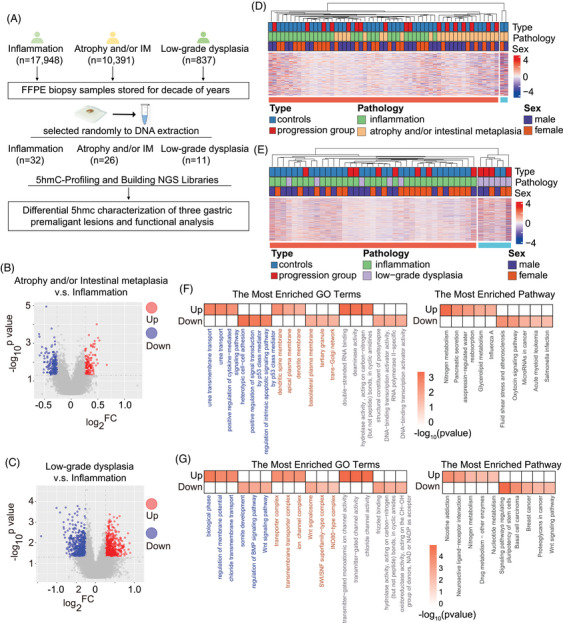
Differential 5‐hydroxymethylcytosines (5hmC) characterization of three gastric premalignant lesions. (A) Differential 5hmC characterization of selected formalin‐fixed paraffin‐embedded (FFPE) samples from three gastric premalignant subtypes for detection. (B and C) Volcano plot showing differentially 5hmC expressed genes in atrophy and/or intestinal metaplasia versus inflammation, low‐grade dysplasia versus inflammation. (D and E) Hierarchical clustering of differentially hydroxymethylated features (false discovery rate [FDR] < 0.05 and fold change >20%) in atrophy and/or intestinal metaplasia versus inflammation, low‐grade dysplasia versus inflammation. (F and G) The KEGG pathway analysis and the Gene Ontology (GO) enrichment analysis of 5hmC in gene bodies that are differentially modified in atrophy and/or intestinal metaplasia versus inflammation, low‐grade dysplasia versus inflammation.

Given the variations in carcinogenic processes across different gastric pathological types, we further investigated their associated 5hmC features. A total of 69 samples from premalignant lesions were selected for genome‐wide 5hmC profiling, 32 patients with inflammation, 26 with atrophy and/or IM and 11 with low‐grade dysplasia (Figure 6A). Further analyses were matched for gender and age, as we observed potential age and gender differences between the different pathological groups (Figure S5A,B). The summarized genome‐wide 5hmC profiles showed enrichment in genomic regions, particularly gene bodies between the TSS and TES, relative to their flanking regions, and depletion at promoter regions around the TSS significantly co‐localized with the enhancer markers H3K4me1 and H3K27ac (Figure S5C). Altered 5hmC modifications specific to atrophy and/or IM and low‐grade dysplasia were identified by individually comparing them to the group of patients with inflammatory gastric lesions (i.e., atrophy and/or IM vs. inflammation; low‐grade dysplasia vs. inflammation). The volcano plot indicated differential hydroxymethylation of the 5hmC features for the two comparisons (Figure 6B,C). Using DESEq2, 232 gene bodies were found to be differentially modified between atrophy and/or IM and inflammation (|log_2_FoldChange| > 0.26, empirical *p*‐value < 0.05), with 102 hyper‐hydroxymethylated and 130 hypo‐hydroxymethylated relative to the inflammation group. Using the same criteria, we identified 823 differentially modified gene bodies (411 hyper‐hydroxymethylated and 412 hypo‐hydroxymethylated) in low‐grade dysplasia compared to the inflammation group. The hierarchical clustering of samples based on the identified differential 5hmC features between atrophy and/or IM and inflammation as well as between low‐grade dysplasia and inflammation by different genomic feature types is shown (FDR < 0.05, fold‐change >20%) (Figure 6D,E).

Functionally, the 232 differential gene bodies between atrophy and/or IM and inflammation were enriched in such KEGG pathways as nitrogen metabolism and pancreatic secretion, which are implicated in gastric mucosal injury, repair and precancerous lesions (Figure 6F).^48^, ^49^, ^50^ In comparison, the 823 differential gene bodies between low‐grade dysplasia and inflammation were enriched with such KEGG pathways as the Wnt signalling pathway, signaling pathways regulating pluripotency of stem cells, proteoglycans in cancer that are involved in cell proliferation, maintenance of stem cell properties and cancer (Figure 6G).^51^, ^52^, ^53^, ^54^

## 4 DISCUSSION

Premalignant lesions are critical indicators of potential cancer development, and their effective monitoring is essential for preventing malignant diseases. How, current surveillance methods for detecting these lesions in GAC are not highly effective. In this study, we conducted a longitudinal survey of gastric pemalignant lesions in Chinese individuals with a median follow‐up time of 12 years. Additionally, by utilizing 5hmC‐Seal assay, we explored a clinically relevant predictive model. This model is expected to lay the foundation for more individualized and effective monitoring of high‐risk individuals, playing an important role in early intervention for GC prevention.

The Correa cascade outlines the multistep progression of gastric carcinogenesis, starting from atrophic gastritis and moving through IM and dysplasia to adenocarcinoma, ultimately leading to GAC.^55^ In our cohort, we combined atrophic gastritis with IM for observation and analysis to gain insights into the development of GAC. A total of 240 cases of GAC were reported during the follow‐up period, with an overall incidence of 0.82%; the median times of inflammation, atrophy and/or IM and low‐grade dysplasia to the development of GAC were 6.68, 6.14 and 4.11 years, respectively, after excluding cases within the first year. Lesions with a suspicious endoscopic appearance require close endoscopic follow‐up, such as prompt repeat endoscopy with multiple biopsies or surveillance at short intervals if pathology is negative (e.g., every 3 months). This emphasis stems from our observation that most GAC cases were diagnosed within the first year of the index endoscopy. A Dutch nationwide cohort study found that a incidence rate of 0.72 per 1000 person‐years for IM and 7.67 per 1000 person‐years for low‐grade dysplasia, with median progression times of 6.1 and 2.6 years, respectively.^56^, ^57^ The observed rates of malignant transformation in our Chinese cohort were relatively higher at 10.8 per 1000 person‐years for atrophy and/or IM and 37 per 1000 person‐years for low‐grade dysplasia (Table 2), likely owing to the higher incidence of GAC among East Asian populations. The SIRs were 2.4, 4.8 and 17 for inflammation, atrophy and/or IM and low‐grade dysplasia, respectively, when compared with the general GAC population in China, indicating a substantially increased risk of GAC in patients with premalignant conditions.

The Chinese clinical guidelines recommend varying surveillance intervals based on lesion types, but adherence faces challenges within the healthcare system. The National Digestive System Disease Clinical Medical Research Center (Shanghai) conducted a big data, multicentre clinical study involving more than 120 hospitals in China in 2019.^58^ A new cancer screening scoring system called Li‐Q score was established, including five variables (age, gender, Hp antibody detection, serum pepsinogen I/II ratio and gastrin 17), with a total score of 23 points (AUC = 83.7%, sensitivity = 63.60% and specificity = 91.10%).^59^ But this GC screening score focuses on screening for GC and does not address precancerous lesions. Our study also demonstrated that age and gender are additional risk factors for GAC development in patients with premalignant gastric lesions, highlighting the necessity for personalized risk assessment. Notably, we constructed an exploratory study of GAC prediction model based on age, gender and pathological type, with an AUC of 72.7% (69.2% sensitivity and 67.8% specificity) (Figure S1), thus underscoring the importance of multi‐factor prediction. However, further research will be necessary to identify additional risk markers to improve patient stratification. Given the importance of epigenetics, we further investigated the 5hmC changes in malignant lesions of gastric.

Epigenetic modifications, including changes in 5hmC, are known to play pivotal roles in cancer biology. Our previous studies and others have highlighted the diagnostic potential of 5hmC modifications in various cancers.^25^, ^60^, ^61^, ^62^ We used the 5hmC‐Seal to establish the first 5hmC Human Tissue Map.^19^ The 5hmC‐Seal technique targets a biologically relevant epigenetic marker 5hmC, and due to its chemical labelling nature, this technique is extremely sensitive, suitable for limited clinical biospecimens and future non‐invasive biomarker discovery (e.g., blood based). Compared to whole‐genome bisulphite sequencing, the 5hmC‐Seal technique is much more cost‐effective, requiring less sequencing depth while still providing meaningful data on 5hmC distribution. Nonetheless, there are also some limitations with the technique, for example, it focuses only on 5hmC and does not provide a comprehensive overview of other modified cytosines, and there may be biases in sample processing that affect results. Our systematic investigation into 5hmC modifications in patient samples provided novel insights into the epigenetic variations associated with the progression of lesions in GC. This underscores the importance of exploring 5hmC's role in GAC and its potential as a biomarker for early detection and a therapeutic target.

Importantly, we also demonstrated the feasibility of a predictive model for GC progression based on 5hmC, which integrates genome‐wide 5hmC profiling with relevant clinical variables such as age, gender and gastric lesion type. Such a predictive tool holds promise for enhancing risk stratification, personalized cancer prevention and early detection strategies. While the model's current performance was promising, future investigations would be benefited by considering, for example, the integration of additional epigenetic marks (e.g., 5mC) as well as genetic mutations to provide a more comprehensive view of the underlying molecular mechanisms for GAC development.

Our study had several limitations. Firstly, there lacked information on factors such as *H. pylori* infection, smoking history and alcohol consumption, all of which can increase the risk of gastric malignancy. Secondly, a standardized biopsy protocol was not employed, potentially leading to varying sensitivities for detecting precancerous lesions due to differences in the number and location of gastric biopsies. However, conducting a study using a standardized biopsy protocol in a large population may not be feasible in the short term given the diversity of endoscopic indications and absence of a widely adopted biopsy protocol. Instead, our data reflected real‐world scenarios wherein clinicians need to make surveillance decisions based on pathology results obtained from imperfect settings. Thirdly, our GAC precursor lesion prediction model, developed with a relatively small sample, though promising, will require further validation and refinement in a larger clinical study. Finally, the lack of other molecular data (e.g., RNA‐seq) on the same samples limited our ability to infer, for example, re‐wired epigenetic pathways related to transcriptomic changes.

In conclusion, the current study provided significant and improved insights into risk stratification among individuals with premalignant gastric lesions, supporting close surveillance for low‐grade dysplasia, particularly in males and older adults. Surveillance endoscopy every 3–6 years may be reasonable for populations at increased risk, such as older patients and patients with extensive IM and/or atrophy, although the optimal surveillance intervals should be further assessed in future studies. Importantly, this study, although limited by the relatively small sample size for genome‐wide 5hmc data, introduced the 5hmC modifications to a clinically relevant predictive model, laying the foundation for individualized more effective monitoring of high‐risk individuals. Hence, expanding sample sizes in future investigations is warranted, with potential implications for targeted strategies in preventing and detecting early‐stage GAC, ultimately improving patient outcomes. Given the favourable prognosis associated with early GAC, our future research endeavours will be dedicated to identifying high‐risk populations, implementing rigorous monitoring protocols and ensuring the timely detection of early detection and treatment GAC in our relentless pursuit of conquering this disease.

## AUTHOR CONTRIBUTIONS

Jie Liu, Wei Zhang, Feifei Luo and Zhongguang Luo contributed to the conception and design of the study. Zhongguang Luo, Wenshuai Li, Wanwei Zheng, Kaiyi Fu, Xiangyu Guo, Bin Lv, Shaocong Mo and Hongyang Zhang contributed to acquisition of data. Zhongguang Luo, Wenshuai Li, Wanwei Zheng, Maolin Ye, Kaiyi Fu, Xiangyu Guo, Shaocong Mo, Yixiang Shi, Bowen Wang and Wei Zhang contributed to analysis of data. Zhongguang Luo, Wenshuai Li, Chuan He and Wanwei Zheng contributed to interpretation of the results. All authors reviewed the manuscript, approved the final version, and agreed to publish this report. All authors had full access to the data appropriate to their roles, and all authors had final responsibility for the decision to submit the manuscript for publication.

## CONFLICT OF INTEREST STATEMENT

Chuan He is a scientific founder, a member of the scientific advisory board and equity holder of Aferna Bio, Inc. and AccuraDX Inc., a scientific co‐founder and equity holder of Accent Therapeutics, Inc., and a member of the scientific advisory board of Rona Therapeutics. Wei Zhang is a consultant for the biomarker discovery program of Tempus Labs, Inc. All the other authors declare no conflict of interest.

## ETHICS APPROVAL

These studies were approved by the Ethics Committee of Huashan Hospital, Fudan University, China (No. 2022–913).

## Supporting information



Supporting Information

Supporting Information

## Data Availability

The data that support the findings of this study are available from the corresponding author upon reasonable request.
