# Supplementary material for: Elucidating epigenetic landscape of gastric premalignant lesions through genome‐wide mapping of 5‐hydroxymethylcytosines: A 12‐year median follow‐up study
Source: Clin Transl Med. 2024 Dec 3;14(12):e70114. doi: 10.1002/ctm2.70114 (PMC11613102; doi:10.1002/ctm2.70114)
Supplement: Supplementary file 1 — Supporting Information [file CTM2-14-e70114-s002.docx]

**SUPPLEMENTARY FIGURE LEGENDS**

**S Figure 1.** A gastric cancer prediction model based on age, sex, and pathological type, with an AUC of 0.727 (sensitivity: 0.692, specificity: 0.678).

**S Figure 2. Cumulative incidence of GAD in patients with premalignant gastric lesions with 10 years follow-up.** The Kaplan–Meier method was used to plot cumulative incidence of gastric adenocarcinoma.


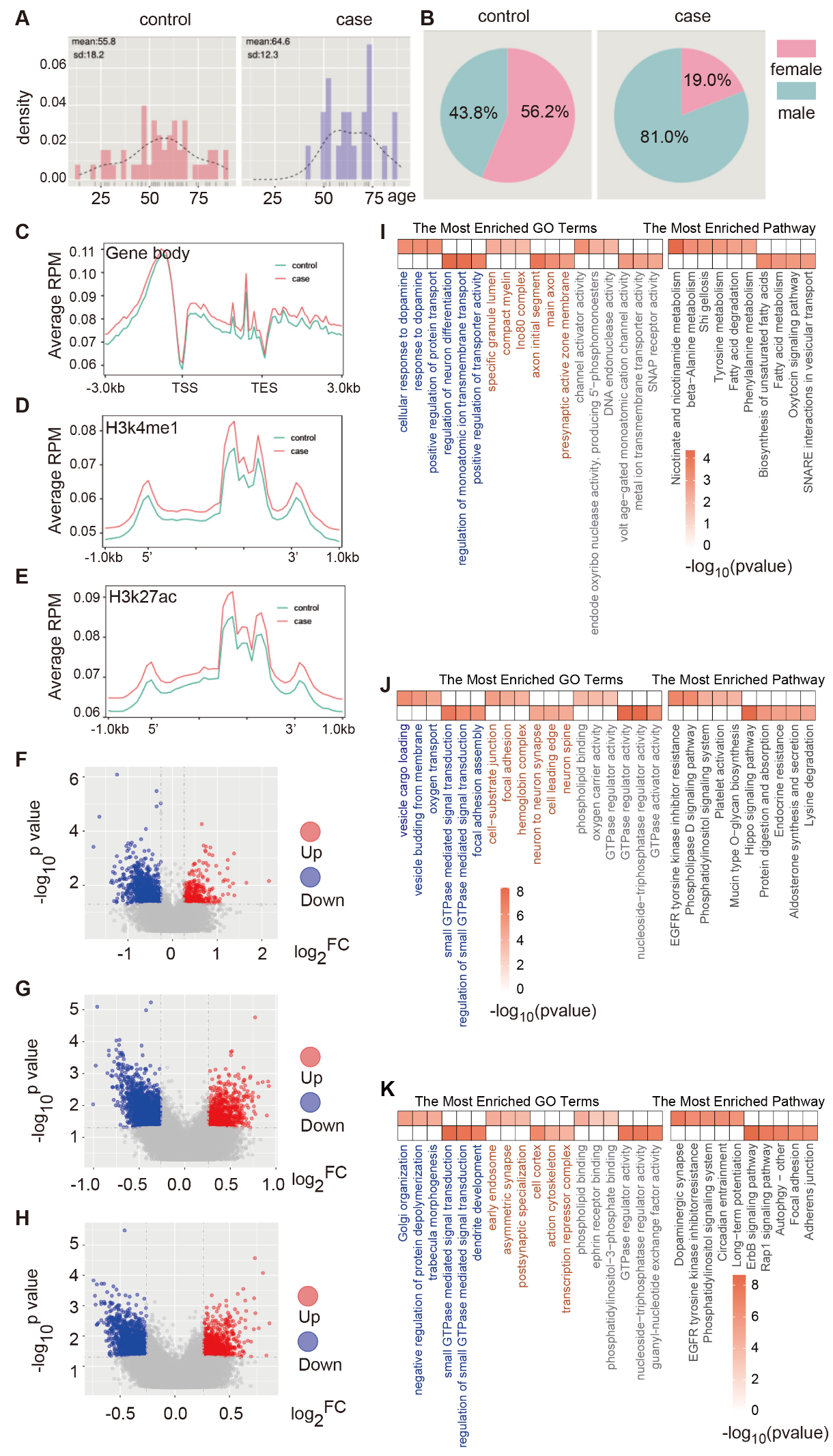


**S Figure 3.** A, B. Potential age and sex differences between 69 samples in progression group vs control group. C, D, E. The 5hmC profiles are distinctly distributed across various genomic feature types in progression group vs control group. F, G, H. The Volcano plots show the differential hydroxymethylation between progression group vs control group in the promoter (F), H3K4me1(G), and H3K27ac(H) regions. I, J, K. The GO enrichment analysis and the KEGG pathway analysis of 5hmC in the promoter(I), H3K4me1(J), and H3K27ac(K) regions between progression group and control group.

**S Figure 4.** The RNA expression of CXCL6, E2F5, GCNT3, and B3GNT3 are highly expressed in gastric cancer patients according to TCGA.


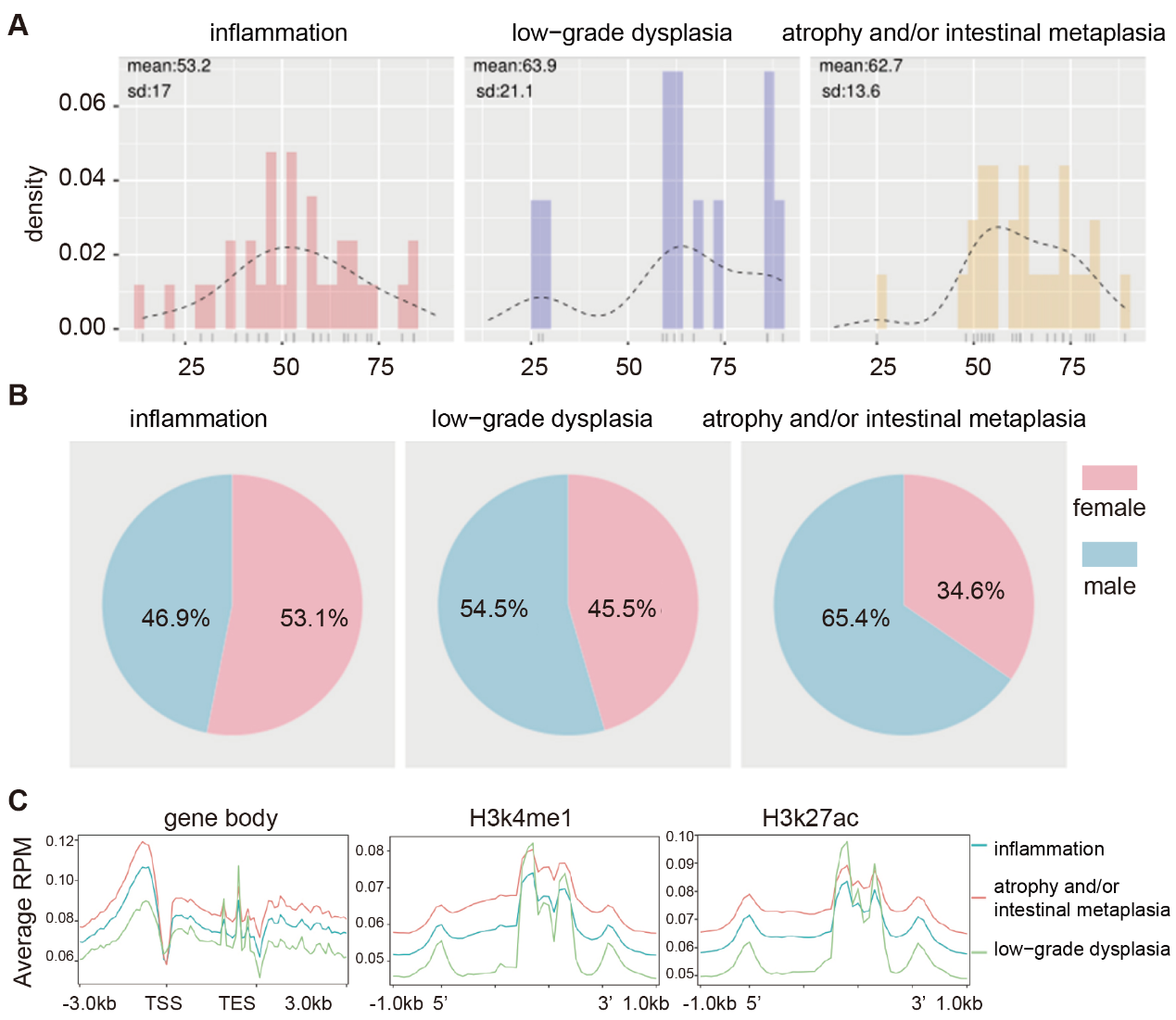


**S Figure 5.** A, B. Potential age and sex differences between 69 samples in different pathological groups. C. The 5hmC profiles are distinctly distributed across various genomic feature types in different pathological groups.

**Supplementary Table 1. Follow-up of patients with gastric inflammation, atrophy and/or intestinal metaplasia and dysplasia diagnosis of gastric adenocarcinoma**

| Number of patients  16198, n (%) | Inflammation  9922(%) | Atrophy and/or Intestinal metaplasia  5808(%) | Low-grade Dysplasia  468(%) |
| --- | --- | --- | --- |
| Total Number of gastric adenocarcinomas, n  51 (0.31) | 20(0.20) | 23(0.40) | 8(1.71) |
| Age at diagnosis of adenocarcinoma |  |  |  |
| Median | 53 | 67 | 71 |
| Interquartile range, years | 29-82 | 49-91 | 54-86 |
| Time to diagnosis of gastric adenocarcinoma (all cases), years |  |  |  |
| *Median* | 3.3 | 2.2 | 0.3 |
| *Interquartile range, years* | (0.1-8.4) | (0.2-9.2) | (0.1-2.5) |
| Sex |  |  |  |
| *Male* | 17(0.17) | 15(0.26) | 4(0.85) |
| *Female* | 3(0.03) | 8(0.14) | 4(0.85) |
| Age in diagnosis of GAD (years) |  |  |  |
| *<50* | 5(0.05) | 1(0.17) | 0 |
| *50-59* | 6(0.06) | 7(0.12) | 1(0.21) |
| *60-69* | 7(0.07) | 5(0.09) | 3(0.64) |
| *70+* | 2(0.02) | 10(0.17) | 4(0.85) |

**Supplementary Table 2. Risk factors for progression to gastric adenocarcinoma among patients with gastric premalignant lesions**

|  | Univariate |  | Multivariate |  |
| --- | --- | --- | --- | --- |
|  | HR (95%CI) | P value | HR (95%CI) | P value |
| Sex  *Male* | 1 |  | 1 |  |
| *Female* | 0.250 (0.137-0.457) | <0.001 | 0.308(0.167-0.567) | <0.001 |
| Age (years) |  |  |  |  |
| *<50* | 1 |  | 1 |  |
| *50-59* | 0.858(0.359-2.048) | 0.730 | 0.836(0.349-2.005) | 0.689 |
| *60-69* | 2.455(1.153-5.228) | 0.02 | 1.995(0.927-4.293) | 0.077 |
| *70+* | 4.683(2.278-9.628) | <0.001 | 3.174(1.512-6.664) | 0.002 |
| Histopathology |  |  |  |  |
| *Inflammation* | 1 |  | 1.0 |  |
| *Atrophy AND Intestinal metaplasia* | 1.992(1.094-3.627) | 0.024 | 1.657(0.899-3.054) | 0.106 |
| *Dysplasia* | 9.001(3.964-20.439) | <0.001 | 6.033(2.614-13.924) | <0.001 |
| Location of intestinal metaplasia |  |  |  |  |
| Fundus | 1 |  |  |  |
| Non-fundus | 0.049(0.000-9920023) | 0.758 |  |  |
